# Strong Impact of Temporal Resolution on the Structure of an Ecological Network

**DOI:** 10.1371/journal.pone.0081694

**Published:** 2013-12-04

**Authors:** Claus Rasmussen, Yoko L. Dupont, Jesper B. Mosbacher, Kristian Trøjelsgaard, Jens M. Olesen

**Affiliations:** 1 Department of Bioscience, Aarhus University, Aarhus C, Denmark; 2 Section of Terrestrial Ecology, Department of Biology, University of Copenhagen, Copenhagen K, Denmark; University of Northampton, United States of America

## Abstract

Most ecological networks are analysed as static structures, where all observed species and links are present simultaneously. However, this is over-simplified, because networks are temporally dynamical. We resolved an arctic, entire-season plant-flower visitor network into a temporal series of 1-day networks and compared the properties with its static equivalent based on data pooled over the entire season. Several properties differed. The nested link pattern in the static network was blurred in the dynamical version, because the characteristic long nestedness tail of flower–visitor specialists got stunted in the dynamical networks. This tail comprised a small food web of pollinators, parasitoids and hyper-parasitoids. The dynamical network had strong time delays in the transmission of direct and indirect effects among species. Twenty percent of all indirect links were impossible in the dynamical network. Consequently, properties and thus also robustness of ecological networks cannot be deduced from the static topology alone.

## Introduction

Today, much community ecology revolves around one of its newest tool kits, complex network analysis, e.g. [1]. Most research in this field uses data aggregated over extensive spatial and temporal spans. In studies of pollination networks, for example, study plot size and period differ by 2–3 orders of magnitude, from just 130 m^2^ to 30 ha and from only three days to as much as 12 years [2]–[3]. This pooling of data in space and time, beyond the range and lifespan of most species and individuals, produces pivotal insight and is clearly necessary in order to obtain robust patterns, but may also blur our understanding of detailed processes behind biodiversity dynamics and preservation. To what extent this is a problem we hardly know [1]. Here, we focus upon this issue and estimate how central properties of a temporally well resolved network are affected when shifting from a static to dynamical analysis, e.g. [4]–[5].

Mutualistic plant–animal networks, and in particular pollination networks, are well–studied in network ecology [2], [6]–[9]. They are 2–mode networks of interacting plants and animals, and their pattern of links shows distinct structures, especially nestedness seems almost ubiquitous [10]–[12]. In a nested pattern we get a link–dense core and two tails of links, because links of specialized species (here tail species) are subsets of links of generalised species (here core species) (File S1). Theoretical studies demonstrate that nestedness stabilises a network against perturbations, again holding implications for conservation, e.g. [13]–[16]. The temporal dynamics of nestedness, however, has rarely been explored [17].

The consensus achieved about structural robustness of static networks in general, is that highly linked hubs are key stabilizers [18]. However, this conclusion was reached without incorporating information about temporal dynamics. Doing so, Tanaka *et al*. [19] demonstrated theoretically that important key nodes, which determine dynamical robustness, got low connectivity. Thus, knowledge about static networks cannot always be extrapolated to their dynamical equivalents [5].

Ecological networks consist of nodes with their inherent temporal dynamics, *e.g.* species have a phenophase, representing their network membership period. During its phenophase, a species interacts with other species, which are entering and leaving the network. A study of such time correlations and the relative temporal ordering of linkage events among species require temporally well–resolved data [20]. In pollination biology and in ecology in general, only few studies are based on datasets partitioned into successive time slices. However, such data make it feasible to track the seasonal or yearly dynamics of networks and their species in detail [2], [21]–[27].

We examined dynamical aspects of a temporally and taxonomically highly resolved arctic plant-flower visitor network, and (1) addressed to what extent the observed nestedness pattern in the static network version, especially the characteristic link tails, but also the link core, was affected by temporal resolution, (2) analysed the temporal dynamics of the nestedness tails in relation to the natural history of their species, and (3) estimated the time delays in the transmission of effects between all species pairs.

## Materials and Methods

### Study site and period

Study site was a 500×500 m plot near the high–arctic research station Zackenberg in NE Greenland (74°28′ N, 20°35′ W) [27]–[29]. Study period included two full seasons (2010 and 2011), *i.e.* from the last snow melted in spring to the first frost and snowfall in autumn. The study site is the same as in [27]. The Greenland Ecosystem Monitoring Coordination Group at the National Environmental Research Institute, Aarhus University approved our research proposals for access and research activities in both 2010 and 2011. No species protected by national or international treaties were sampled in this project. For both years, we constructed a plant-flower visitor network of all flowering plant species and their flower visitors (here operationally termed pollinators). The ‘network phenophase’ of a plant species is the time between its first and last observed pollinator visit, and the network phenophase of a pollinator species is the time between its first and last observed visit to a flower. If ≥1 interaction was observed between a plant and a pollinator species they were scored as interacting during their entire phenophase overlap. This is the most conservative approach with respect to the estimation of visitation activity and hides single days of inactivity during their phenophase. The complete network season for the study site lasts from when the first to the last flower of any plant species is observed to receive a visit from any pollinator species. In 2010 and 2011, the network seasons lasted 70 and 69 days, and 54 and 52 days were spent observing and collecting in the field, respectively. All field days were sunny and calm, and thus assumed being suitable for foraging insects.

### Pollinator census

On each field day lasting from 09 to 17 hrs, observations of insect visitation to flowers were made at all flowering plant species within the study plot by CR or JBM. The daily census per plant species lasted 40 min, *i.e.* we spent 20 min at each of two randomly selected flowering individuals (same observation protocol as in [27]). If single plant individuals were impossible to discern, we defined an individual as a square of 5 cm×5 cm plant cover. Thus total seasonal observation time of a plant species was 40 min x phenophase length (days). Most insect visitors could not be identified in the field, but were collected and later identified by specialists. Representatives of all identified species were also barcoded to confirm taxonomical affiliation.

### Phenophase of core and tail species

Pollination generalization level of a species *i* was given as its linkage level *L_i_*, which is the total number of links of *i* to other species during the entire season, *i.e. L* is a static network property. For simplicity, we sorted all species into tail and core species with *L*≤2 and >2, respectively (File S1; [2], see also [29]).

### Nestedness tail

We constructed a static version of our study network by pooling all species and links for an entire season. Using our daily visitation observations and the definition of network phenophase of a species (see *Study site and period*), we also described the network temporally as a consecutive series of networks, each representing one field day, *i.e.* each network had a slice “thickness” of only one day. We visualized the temporality of the network during an entire season using the R library ‘timeordered’ by Blonder *et al*. [30].

Level of nestedness ‘*NODF*’ (Nestedness measure based on Overlap and Decreasing Fills) of the static and temporal versions was estimated using the software ANINHADO v. 3.03 [31]–[32]. Number of tail ‘*t*’ species and core species ‘*c*’ of the temporal and static network was compared.

In a nested network, tail species are linked to core species, and most pollination networks have relatively many tail pollinator species compared to tail plants [33]. We tested if the phenophase of tail pollinator species visiting the same core plant species segregated randomly in time. That is, if the tail pollinators were linked and interacted in a predictable manner to the core plant species in the network or not. This is a so–called 1–dimensional “pencil box” or mid–domain effect [34], where most overlap under random expectations is expected to be around the middle of the season. The ‘domain’ becomes the time-span from the phenophase start of the earliest tail pollinator species to the end of the last pollinator tail species of a given core plant species. The software *RangeModel* v. 5 tests if the observed segregation of tail pollinator phenophases across the domain or the season of the core plant differs from random [35]. *RangeModel* is a Monte Carlo simulation tool for assessing geometric constraints on species richness.

### Time delay between species in the network

**Figure 1:**
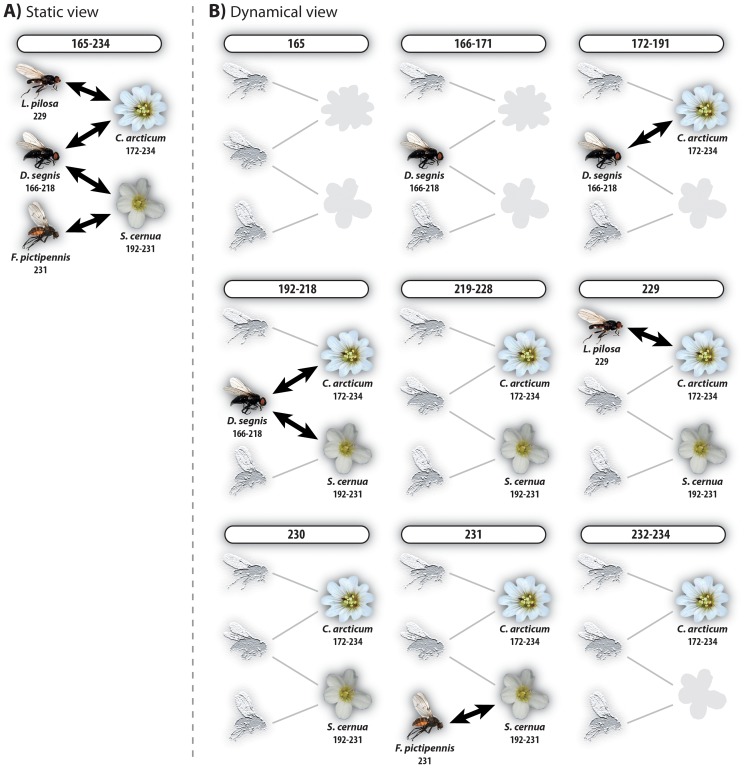
A five–species subnetwork of the Zackenberg pollination network, 2010, including three pollinator species and two plant species. The 2010–season lasted from day 165 to day 234. Numbers below a species gives its phenophase. A, static view: All species interact directly or indirectly, e.g. *Cerastium arcticum* interacts directly with *Lasiopiophila pilosa* and *Drymeia segnis*, and indirectly with *Saxifraga cernua* and *Fucellia pictipennis*. B, dynamical view: The season is cut into nine temporal windows as defined by the phenologies of the species, capturing the different linkage events. Bars above each time-window display the length of the time-frame. Within a given time window, highlighted species and links are active, whereas faded ones are inactive, *i.e.* they are either active earlier or later.

**Figure 2:**
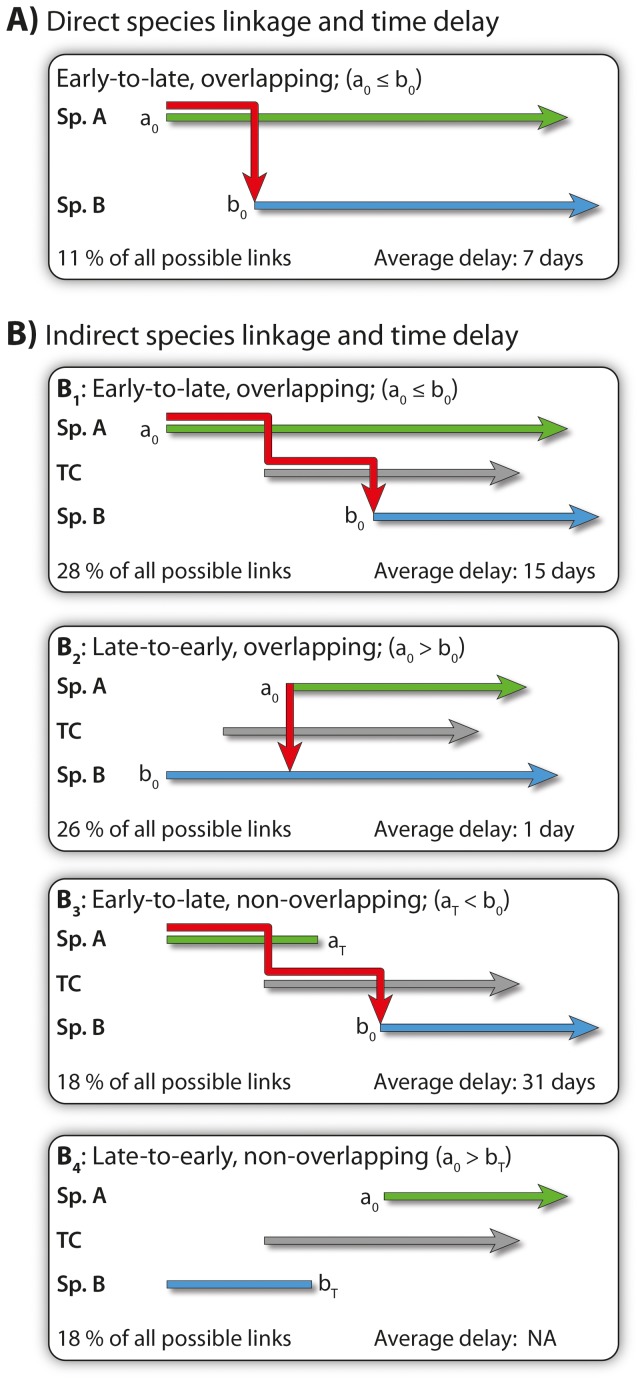
Direct (A) and indirect (B) species linkage and time delays (days) between species pairs. Phenophases of species pairs are given as green and blue arrows, and linkage is here directed from green to blue. Red arrows show the temporal linkage path. Four kinds of indirect linkage are shown. In the first two, species phenophases are overlapping, whereas in the latter two they do not. Percentages are total sums for all 1– and 2–mode networks. a_0_ and b_0_ are starting dates of pollinator species A and B, respectively, whereas a_T_ and b_T_ are ending dates. TC's are temporal coupler species.

In an ecological network all species are connected, either directly or indirectly (Figs. 1–2). If the latter is the case, the connection passes through a series of directly linked species. The time delay (or distance) *d*_ij_, between two directly or indirectly linked species *i* and *j* is the time difference between the start of their phenophases. *i* and *j* may be a plant and pollinator species, two plants or two pollinators. In the latter two cases, the linkage is always indirectly through either a pollinator or a plant, respectively. Such species become temporal couplers of *i* and *j* (TC in Fig. 2B). In order to estimate *d*_ij_, we used the phenophase data. Then the delay between any species pair has to be constrained by the relative position of their phenophases [20]. In [20], Tang *et al*. looked at 1–mode human–contact and brain–cortical networks, but the concept can easily be adopted by 2–mode network analysis. A 2–mode network is made up of two interacting communities, e.g. plants and their pollinators. Static ecological networks are most often small–worlds with short path length and high clustering, resulting in high connectivity, e.g. [36]. In such networks, species and their links are all assumed to have complete temporal overlap, *i.e.* their presence is simultaneous and *d*_ij_,  = 0 (Figs. 1A, 2A). Consequently, disturbances spread immediately among species, whether they are directly or indirectly connected, because time delays are ignored. Thus static networks overestimate real connectivity because they do not catch these time–dependent properties [20]. This crucial difference between static and dynamic networks is illustrated in Figs. 1–2.

In addition to phenophase, other natural history constraints affect time delay between indirectly liked species, e.g. variation in host plant preference. Here, such transmission delay effects caused by the specific biology of the temporal couplers were ignored.

*d*_ij_ was calculated for all species pairs and related to network type, *i.e.* 2–mode directed networks (plant–to–pollinator network and pollinator–to–plant network) and derived 1–mode networks (pollinator–pollinator and plant–plant networks). We also calculated mean <*d*> between directly or indirectly linked core and tail species.

## Results

### Phenophase of core and tail species

Core plants and pollinators had similar phenophase length (30–33 days; Table 1; Wilcoxon test [2010–2011 pooled]: *Z* = 0.056, *P* = 0.96) and so had tail plants and pollinators (4–8 days; Table 1; Wilcoxon test [2010–2011 pooled]: *Z* = 1.42, *P* = 0.16). Core plants, however, had a seven times longer phenophase than tail plants, and core pollinators had a four times longer phenophase than tail pollinators (Table 1). Phenophase length correlated with *L* (Plants [2010–2011 pooled]: *R* ^2^ = 0.71, *F*_1.64_ = 160, *P*<0.001; pollinators: *R* ^2^ = 0.59, *F*_1.164_ = 239, *P*<0.001).

**Table 1:** Comparison of static and dynamical networks.

Season	Start (d)	End (d)	Length (d)
2010	165	234	70
2011	167	235	69

Average phenophase (d)	Pollinators	Plants
2010		
Core species	32.3	32.5
Tail species	8.1	3.7
2011		
Core species	30.3	30.8
Tail species	8.1	5.1

Networks	Static networks		Dynamical networks	
	Pollinator species	Plant species	Average daily no. pollinator spp.	Average daily no. plant spp.
2010				
No. core species (*c*)	38	27	17.9	12.8
No. tail species (*t*)	40	7	4.8	0.5
Relative tail length *t*/(*c*+*t*)	0.51	0.21	0.21	0.03
Total	78	34	22.73	13.26
2011				
Core species (*c*)	27	24	12.8	11.5
Tail species (*t*)	61	8	7.6	0.6
Relative tail length *t*/(*c*+*t*)	0.69	0.25	0.37	0.05
Total	88	32	20.34	12.16

A core species has L>2 and a tail species has L≤2. L of a species is its number of links to other species. d, day number or number of days.

**Figure 3:**
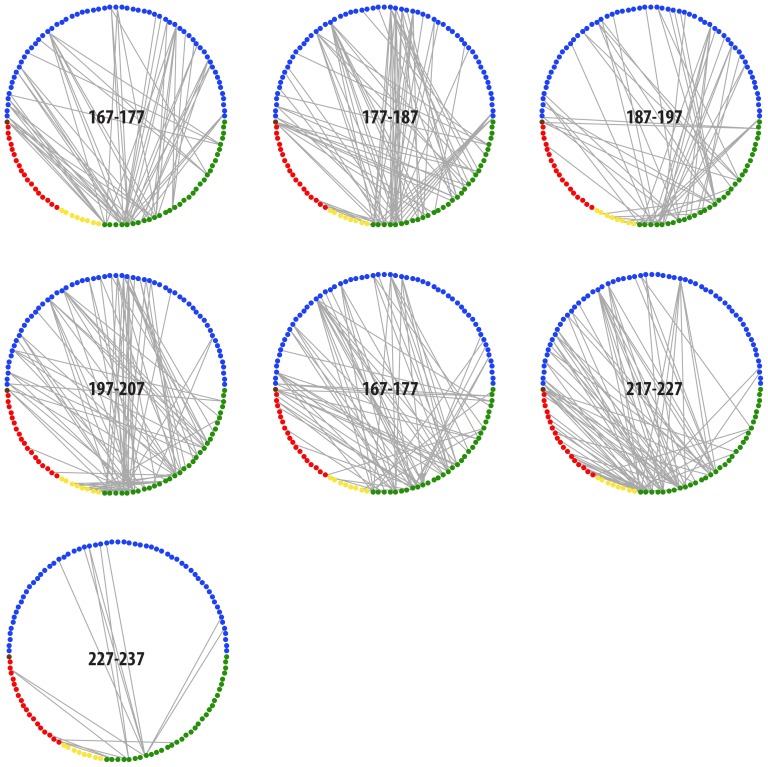
Visualization of the temporal dynamics of the network in 2011. It shows the strong variability in link pattern during the season. Each network is based on the accumulation of data over a 10–day period, with day of year indicated in for each of the seven networks. Green represent the plants and other colors are insects (blue, Diptera; brown, Heteroptera; red, Hymenoptera; yellow, Lepidoptera). The figure was made in the R package ‘timeordered’ [30].

The temporal dynamics of the network is shown in Fig. 3, illustrating its high variability in link density. Here, the individual networks have a time–slice thickness of 10 days.

### Nestedness tails

Static network matrices were significantly nested (2010: *NODF*  = 7.8, *P*<0.05; 2011: *NODF*  = 6.3, *P*<0.01).

We compared tail length of static and dynamical 1–day networks. In static networks, relative tail length [*t*/(*c*+*t*); where *t* and *c* are number of tail and core species, respectively] was 0.51–0.69 for pollinators and 0.21–0.25 for plants (Table 1). Thus, the static networks had more tail pollinator species than core species (*t*/(*c*+*t*)>0.50), whereas overall only a few plants were tail species. In the dynamical networks, however, the daily tails got stunted: *t*/(*c*+*t*) = 0.21–0.37 for pollinators and only 0.03–0.05 for plants (Table 1). Consequently, a distinct nestedness tail only became discernible in networks with increasing temporal data pooling. Only a mean of 5–8 daily tail species were present simultaneously in the dynamical 1–day networks in contrast to 40–61 tail species in the static network (Table 1).

**Figure 4:**
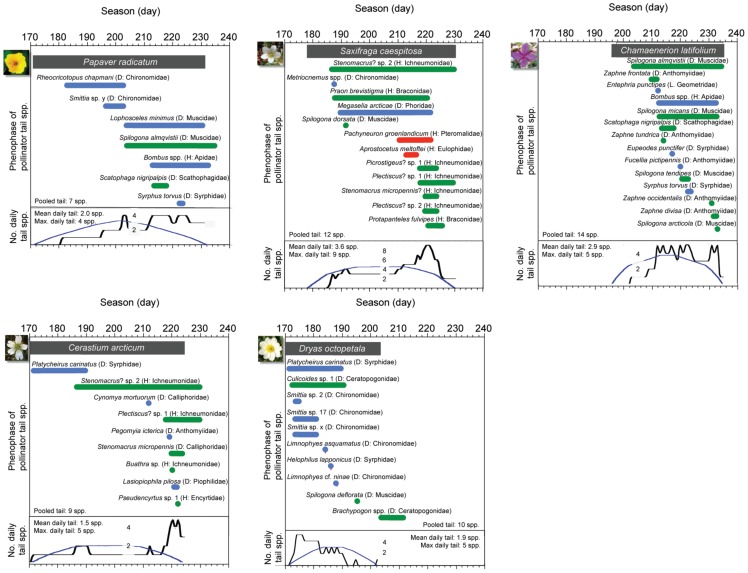
Temporal segregation of tail pollinators for core plants with the longest tail. Black bars indicate plant phenophase and blue, green and red those of tail pollinators, flower visiting parasitoids and flower visiting hyper–parasitoids, respectively. The black curve at the bottom of each panel gives the daily variation in number of tail species to the particular core plant, whereas the blue curve gives the expected number under random conditions as produced by the software *RangeModel*.

Temporal segregation of tail pollinator species for each core plant species differed significantly from random (File S2, data from 2011), and each core plant had a late–seasonal burst of tail pollinators (Fig. 4). The core plant *Dryas octopetala* differed from this pattern, because it had an early burst of tail pollinators.

### Time delay

We analysed time delay *d* among species (Table 2). In the 2010–network, total numbers of pollinator and plant species were *A = *78 and *P* = 34 species, respectively, and link number was *I* = 295, giving a connectance of *C* = 100 *I/*(*AP*) = 11% for the static network. In the 1–day networks, effects between the 295 linked species pairs were on average delayed 6–8 days (Table 2A). Since only 11% of all possible direct links were observed, 89% remained unobserved or forbidden [28]. However, since the static network was one coherent giant component, unlinked species pairs had to be indirectly connected through other species termed static couplers. Indirect linkage in dynamical networks was more complex. These networks had four kinds of indirect linkage (Fig. 2B): two kinds connected species with overlapping phenophases, and two kinds connected species with non–overlapping phenophases. Indirect links passed through temporal coupler species (TCs), *e.g.* indirect links between plants passed through pollinator couplers and indirect links between plants and pollinators passed through 1–several pairs of pollinator–plant couplers (Fig. 2). Indirect links between species with overlapping phenophases were more frequent than between those with non–overlapping phenophases (60% *vs*. 40%; Table 2D). Indirect links between species with overlapping phenophases and where the connection went from the earliest to the latest species had a mean delay of 15 days (Table 2D). Indirect links between species with overlapping phenophases and where the connection went from the latest to the earliest species had no delay, *i.e.* 1 day (Table 2D), because when the late species entered the network it could immediately interact through couplers with the early species already present in the network. Indirect links between species with non–overlapping phenophases and where the link went from the earliest to the latest species had a mean delay of as much as 31 days (Table 2D). Indirect links between species with non–overlapping phenophases and where the link had to pass from the latest to the earliest species were impossible (Table 2D), because a species *i* entering the network after species *j* has left the network cannot connect to *j*, *i.e.* effects back in time were impossible, at least within the same season. Such temporally impossible indirect links constituted 20% of all indirect links (Table 2E), and they are a unique feature of temporally dynamical networks, not discernible in their static equivalents.

**Table 2:** Frequency of direct and indirect species interactions and their time delays.

Direct species linkage and time delay			
A		Delay between linked species (days)	
	From plant to pollinator	8.4	
	From pollinator to plant	6.2	
	Mean delay	7.3	
B	Delay between directly linked species (days)	To core	To tail
	From core	9	25
	From tail	5	19

Indirect species linkage and time delay						
		Overlapping phenophases		Non-overlapping phenophases		
C	Absolute numbers of links	Early-to-late	Late-to-early	Early-to-late	Late-to-early	Total
	From pollinator to pollinator	1740 (29%)	1616 (27%)	1331 (22%)	1319 (22%)	6006
	From plant to pollinator	865 (37%)	623 (26%)	491 (21%)	378 (16%)	2357
	From pollinator to plant	683 (29%)	794 (34%)	379 (16%)	501 (21%)	2357
	From plant to plant	448 (40%)	408 (36%)	134 (12%)	132 (12%)	1122
	Total	3736 (32%)	3441 (29%)	2335 (20%)	2330 (20%)	11842
D	Delay between indirectly linked species (days)	Early-to-late	Late-to-early	Early-to-late	Late-to-early	
	From pollinator to pollinator	15	1	31	_	
	From plant to pollinator	17	1	34	_	
	From pollinator to plant	14	1	28	_	
	From plant to plant	14	1	31	_	
	Mean delay	15	1	31	_	
E	Forbidden linkage (pct.)^1^	To core	To tail			
	From core	5	14			
	From tail	28	40			

1 Non-overlapping, late-to-early interactions

On average, direct linkage time delay was as short as 5 days from a tail species to its interacting core species (Table 2B) and so was the delay between core species (9 days). In contrast, delay from a core species to a tail species was long (25 days), and so was the delay between tail species (19 days). For the indirectly linked species, only 5% of all core–core species connections were temporally impossible (Table 2E). Whereas, the same figure for tail–tail species connections was 40%. Temporally impossible connections from tail to core species were twice as frequent, as in the opposite direction (28% *vs.* 14%; Table 2E).

## Discussion

### Static vs. dynamic networks

Spatio–temporal data accumulation in network studies is essential in order to obtain robust results. However increasing the spatial and temporal scale also blurs our understanding of the finer dynamics, because during accumulation, we artificially increase the incidences of spatial and phenological coupling between species by ignoring the spatio-temporal ordering of the species and their links [28], [37]. Here we compared the structure and behaviour of static and temporally dynamical networks.

First we looked at the iconic pattern of nestedness observed in most 2–mode networks [10]–[12]. Only a few pollination networks are sufficiently resolved in time to allow temporal analyses of nestedness and frequencies of core and tail species [38]. In a 4–yr Greek plant–pollinator network study [25], a 12–yr Spanish nectar plant–butterfly network study [2], and a 4–yr Chinese plant–pollinator study [23], annual turnover of core and tail species was estimated. Only [2] used the core–tail dichotomy, but in essence all studies demonstrated that in contrast to core species, tail species had a high turnover across seasons or years. Here we showed that even within seasons the turnover of tail species was strong.

The generic picture of strong nestedness in static networks, which portrays two tails of many specialists and a core of a few generalists, needs to be reconsidered in dynamical network analysis.

Firstly, the static network had a long tail of pollinators, but a very short plant tail. This may be grounded in methodology as our sampling protocol was plant–focused, *i.e.* we made our observations at flowers and did not follow individual pollinators flying among plants. A plant–focused approach may accumulate more links per plant than per pollinator species. In general, all pollination network studies are plant–focused, except for a couple including information about pollinator pollen load, e.g. [39], and the pollinator tail is, generally, longer than the plant tail. In a sample of 47 pollination networks, relative pollinator tail length (*t*/(*t*+*c*)) was 0.67, but significantly lower for the plant tail, *viz*. 0.29 (Paired *t* = 4.61***; *unpublished*). These figures are similar to the ones from this study, *viz*. 0.69 and 0.25, respectively. In the pollinator–focused study by Bosch *et al*. [39], the pollinator tail shortened 0.6–fold. In 26 seed dispersal network studies from the literature, 13 were animal–focused (data collected from faeces or by tracking foraging animals), five were plant–focused and eight both. Relative tail length of plant–focused networks was 0.36 and 0.19 for animals and plants, respectively. However, in animal–focused networks the plant tail was longest, *viz*. 0.28 and 0.49 for animal and plant tails, respectively. In studies based on both plant and animal sampling, tails were of equal size, *viz*. 0.22 and 0.28 for animals and plants, respectively. Thus the tail was longest for the community, which was not the focus of the observations (Paired *t* = 2.72**; *unpublished*). Therefore the variation in tail length is partly driven by methodology.

Secondly, tail pollinator species make up a mixed bag of ecological and evolutionary specialists: floral reward specialists, pollinator predators and parasitoids, rare species, common species at their range margin, migrants, species including floral resources as a minor dietary component, and arctic and montane animals using flowers as sun–basking sites (*pers. obs.*). However, in spite of this diversity of ecological roles, their network topological role, in general, becomes the same, *viz*. interaction with core network members resulting in asymmetrical dependency between the core and tail [40]–[41]. Static nested networks show long tails connected to a few core species and thus suggest functional and topological redundancy or equivalence among tail species [42]. In dynamic network mode, this interpretation is too simplified and perhaps wrong. In our arctic community the pollinator tail constitute a temporal sequence of ephemeral species, together offering a continuous supply of mutualistic partners to the core plant members. This may be important in sustaining core plants during their long phenophase, *i.e.* closing temporal windows with otherwise insufficient pollinator supply.

Thirdly, towards the end of the season, core plants build–up a small food web in their tail, consisting of pollinators, parasitoids and even hyper–parasitoids. *Dryas octopetala*, however, had a burst of tail visitors at the beginning of its flowering. The reason might be that this plant species was exceedingly abundant and thus acted as a strong attractant to several early–season insects.

### The arctic dominance of Diptera and hymenopteran parasitoids

Compared to low–latitude pollination networks, arctic networks have a unique taxonomic pollinator composition. At Zackenberg, 74% and 14% of the pollinator fauna were dipterans and hymenopteran parasitoids, respectively. Worldwide, Diptera only constitutes 42.0% (*unpublished*) of the fauna of a pollination network. However, the range known from arctic sites is 67–77% [43]–[44]. The diversity of flower–visiting hymenopteran parasitoids may also increase with latitude [45]. Consequently, an understanding of the natural history of these two dominant groups may explain how they drive the topology of arctic networks.

At Zackenberg, most insect groups had representatives in the pollinator tail. Among the hymenopteran parasitoids almost all were tail species (2010: 86%; 2011: 94%). Muscidae and Chironomidae were the richest Diptera families both with many tail species. Chironomidae becomes more dominant in the high Arctic. Greenland has 100+ species [46], and at Zackenberg they constituted 25 out of 108 observed pollinator species. Tail species are rarely abundant [27], but Chironomidae is an exception. At Zackenberg, most were tail species (2010: 9 of 18 spp.; 2011: 11 of 15 spp.) and their abundance is often very high (20–50% of total insect abundance, [47]). The adult life stage of arctic Chironomidae lasts only a few weeks, most adults have reduced biting mouthparts, and their emergence as adults is often very synchronized. Thus the reasons for their occurrence in the tail may be their synchronized short adult life stage and low food intake in adulthood [46] (for other Diptera families, see File S3).

### Conclusions

In summary, our tale of tail goes as follows: The first species to appear in the network at the beginning of the season are those that have the longest phenophase, reach the highest abundance, become generalists and thus act as core species. They become the structural backbone of the network on which biodiversity “hangs” its tails of specialists [27]. Most tail species are only members of the network for a few days, but are succeeded by other topologically equivalent species, *i.e.* tail species turn–over becomes high. Thus the characteristic tails of specialists observed in a traditional portraiture of static networks are much shorter in the temporal view. Consequently, when static network papers talk about core species surrounded by a swarm of specialists it may be more a result of data accumulation, e.g. [48]. The swarm is a rather temporal series of species linking successively to the core. The tail species are important to the core, because if some drop out of the network, their temporal sequence breaks apart, time windows are opened up, maybe forcing core species to leave, and ultimately fragmenting the network.

With increasing global warming, the network season is prolonged, affecting the coupling between core plants and tail pollinators [49]–[51]. If the phenophase of core plants is extended beyond that of pollinators, temporal gaps in the sequence of tail pollinators may appear, affecting temporal network dynamics. This may, particularly, have consequences to late–seasonal pollinators also being members of higher trophic levels, such as parasitoids. Thus dissecting the temporal structure of the tail, adds layers of complexity to our general understanding of network behaviour.

The temporality of the network was analysed by measuring the delay for any generic process spreading “information” between directly or indirectly interacting species. Our estimate informs us about potential delays of mutualistic and antagonistic effects among species. Twenty per cent of all connections in the network were impossible and others first became established after considerable delays. This must strongly affect network properties, *e.g.* robustness. However, we hardly know what we mean when talking about robustness of dynamical networks, but a prerequisite for an analysis are well–resolved data at an adequate scale to the measurement of delays in the transmission of disturbance effects.

## Supporting Information

File S1 **Matrices sorted in a nested way**. Left: A perfectly nested matrix with all links in the shaded area. Right: 88 pollinators are listed in rows and 32 plants in columns (data from 2011). Species are listed according to descending linkage level L from the upper left corner. L of a species is its number of links to other species. If two species have similar L, they are subsequently sorted according to increasing L of their interacting partners. Sixty–one pollinator species (69% of total) and eight of all plant species (28%) constituted the tails. Thus the tail of the pollinator community is much longer than that of the plants.
(TIF)

File S2 **Descriptive statistics from RangeModel analyses**. For each core plant, we made 5,000 Monte Carlo runs, using the phenophases of tail pollinators. Empirical D is average daily difference between observed species number and mean species number from the Monte Carlo runs. Empirical rank is rank of the Empirical D compared to the 5,000 D-values from the runs. Percentile is Empirical rank/5,001. Mean, Min and Max D are average, minimum and maximum of the 5,000 runs.
(DOCX)

File S3 **The importance of Muscidae and Anthomyiidae (Diptera) at Zackenberg**.
(DOCX)

## References

[1] Woodward G, Benstead JP, Beveridge OS, Blanchard J, Brey T, et al (2010) Ecological networks in a changing climate. Advances in Ecological Research 42: 71–138.

[2] Olesen JM, Stephaniscu C, Traveset A (2011) Strong, long-term temporal dynamics of an ecological networks. PLoS ONE 6: e26455. 22125597 10.1371/journal.pone.0026455 PMC3219636

[3] Trøjelsgaard K, Olesen JM (2012) Macroecology of pollination networks. Global Ecology & Biogeography 22: 149–162.

[4] Belgrano A, Scharler UM, Dunne J, Ulanowicz RE, editors (2005) Aquatic food webs: an ecosystem approach. Oxford: Oxford University Press.

[5] Perra N, Gonçalves B, Pastor-Satorras R, Vespignani A (2012) Acitivity driven modeling of time varying networks. Scientific Reports 2: 469. 22741058 10.1038/srep00469 PMC3384079

[6] Bascompte J, Jordano P (2007) Plant-animal mutualistic networks: the architecture of biodiversity. Annual Review of Ecology and Systematics 38: 567–593.

[7] Burkle LA, Alarcón R (2011) The future of plant-pollinator diversity: understanding interaction networks across time, space, and global change. American Journal of Botany 98: 528–538. 21613144 10.3732/ajb.1000391

[8] Hagen M, Kissling WD, Rasmussen C, Aguiar MAMD, Brown LE, et al (2012) Biodiversity, species interactions and ecological networks in a fragmented world. Advances in Ecological Research 46: 89–210.

[9] Olesen JM, Dupont YL, Hagen M, Rasmussen C, Trøjelsgaard K (2012) Structure and dynamics of pollination networks: the past, present and future. In: Patiny S, editor. *Evolution of Plant–Pollinator Relationships.* Cambridge: Cambridge University Press. pp. 374–391.

[10] Bascompte J, Jordano P, Melián CJ, Olesen JM (2003) The nested assembly of plant-animal mutualistic networks. Proceedings of the National Academy of Sciences of the United States of America 100: 9383–9387. 12881488 10.1073/pnas.1633576100 PMC170927

[11] Dupont YL, Hansen DM, Olesen JM (2003) Structure of a plant-pollinator network in the high-altitude sub-alpine desert of Tenerife, Canary Islands. Ecography 26: 301–310.

[12] Ollerton J, Johnson SD, Cranmer L, Kellie S (2003) The pollination ecology of an assemblage of grassland asclepiads in South Africa. Annals of Botany 92: 807–834. 14612378 10.1093/aob/mcg206 PMC4243623

[13] Fortuna M, Bascompte J (2006) Habitat loss and the structure of plant-animal mutualistic networks. Ecology Letters 9: 281–286. 16958893 10.1111/j.1461-0248.2005.00868.x

[14] Bastolla U, Fortuna MA, Pascual–García A, Ferrera A, Luque B, et al (2009) The architecture of mutualistic networks minimizes competition and increases biodiversity. Nature 458: 1018–1020. 19396144 10.1038/nature07950

[15] Thébault E, Fontaine C (2010) Stability of ecological communities and the architecture of mutualistic and trophic networks. Science 329: 853–856. 20705861 10.1126/science.1188321

[16] James A, Pitchford JW, Plank MJ (2012) Disentangling nestedness from models of ecological complexity. Nature 487: 227–230. 22722863 10.1038/nature11214

[17] Nielsen A, Bascompte J (2007) Ecological networks, nestedness and sampling effort. Journal of Ecology 95: 1134–1141.

[18] Albert R, Jeong H, Barabási A-L (2000) Error and attack tolerance of comlex networks. Nature 406: 378–382. 10935628 10.1038/35019019

[19] Tanaka G, Morino K, Aihara K (2012) Dynamical robustness in complex networks: the crucial role of low-degree nodes. Scientific Reports 2: 232. 22355746 10.1038/srep00232 PMC3265565

[20] Tang J, Scellato S, Musolesi M, Mascolo C, Latora V (2010) Small-world behavior in time-varying graphs. Physical Review E 81: 055101. 10.1103/PhysRevE.81.055101 20866285

[21] Alarcón R, Waser NM, Ollerton J (2008) Year-to-year variation in the topology of a plant-pollinator interaction network. Oikos 117: 1796–1807.

[22] Dupont YL, Olesen JM (2012) Stability of modularity and structural keystone species in temporal cumulative plant-flower-visitor networks. Ecological Complexity 11: 84–90.

[23] Fang Q, Huang S-Q (2012) Relative stability of core groups in pollination networks in a biodiversity hotspot over four years. PLoS ONE 7: e32663. 22412902 10.1371/journal.pone.0032663 PMC3297609

[24] Kaiser-Bunbury CN, Muff S, Memmott J, Müller CB, Caflisch A (2010) The robustness of pollination networks to the loss of species and interactions: a quantitative approach incorporating pollinator behaviour. Ecology Letters 13: 442–452. 20100244 10.1111/j.1461-0248.2009.01437.x

[25] Petanidou T, Kallimanis AS, Tzanopoulos J, Sgardelis SP, Pantis JD (2008) Long-term observation of a pollination network: fluctuation in species and interactions, relative invariance of network structure and implications for estimates of specialization. Ecology Letters 11: 564–575. 18363716 10.1111/j.1461-0248.2008.01170.x

[26] Lundgren R, Olesen JM (2005) The dense and highly connected world of Greenland's plants and their pollinators. Arctic, Antarctic, and Alpine Research 37: 514–520.

[27] Olesen JM, Bascompte J, Elberling H, Jordano P (2008) Temporal dynamics in a pollination network. Ecology 89: 1573–1582. 18589522 10.1890/07-0451.1

[28] Olesen JM, Bascompte J, Dupont YL, Elberling H, Rasmussen C, et al (2011) Missing and forbidden links in mutualistic networks. Proceedings of the Royal Society of London, Series B 278: 725–732. 20843845 10.1098/rspb.2010.1371 PMC3030842

[29] Olesen JM, Dupont YL, O'Gorman E, Ings TC, Layer K, et al (2010) From Broadstone to Zackenberg: Space, time and hierarchies in ecological networks. Advances in Ecological Research 42: 1–69.

[30] Blonder B, Wey TW, Dornhaus A, James R, Sih A (2012) Temporal dynamics and network analysis. Methods in Ecology and Evolution 3: 958–972.

[31] Almeida-Neto M, Ulrich W (2011) A straightforward computational approach for measuring nestedness using quantitative matrices. Environmental Modelling & Software 26: 173–178.

[32] Guimarães PR Jr, Guimarães P (2006) Improving the analyses of nestedness for large sets of matrices. Environmental Modelling & Software 21: 1512–1513.

[33] Olesen JM (2000) Exactly how generalised are pollination interactions? The Norwegian Academy of Science and Letters, Oslo. Pp. 161–178.

[34] Colwell RK, Hurtt GC (1994) Nonbiological gradients in species richness and a spurious Rapoport effect. American Naturalist 144: 570–595.

[35] Colwell RK (2006) RangeModel: A Monte Carlo simulation tool for assessing geometric constraints on species richness. v. 5. User's Guide and application. http://viceroy.eeb.uconn.edu/rangemodel).

[36] Olesen JM, Bascompte J, Dupont YL, Jordano P (2006) The smallest of all worlds: pollination networks. Journal of Theoretical Biology 240: 270–276. 16274698 10.1016/j.jtbi.2005.09.014

[37] Hegland SJ, Nielsen A, Lázaro A, Bjerknes A-L, Totland Ø (2009) How does climate warming affect plant-pollinator interactions? Ecology Letters 12: 184–195. 19049509 10.1111/j.1461-0248.2008.01269.x

[38] Nielsen A, Bascompte J (2007) Ecological networks, nestedness and sampling effort. Journal of Ecology 95: 1134–1141.

[39] Bosch J, Martín AG, Anselm R, Navarro D (2009) Plant-pollinator networks: adding the pollinator's perspective. Ecology Letters 12: 409–419. 19379135 10.1111/j.1461-0248.2009.01296.x

[40] Petanidou T, Ellis WN (1996) Interdependence of native bee faunas and floras in changing Mediterranean communities. In: Matheson A, Buchmann SL, O'toole C, Westrich P, Williams IH, editors. The Conservation of Bees. London: Academic Press. pp. 201–226.

[41] Bascompte J, Jordano P, Olesen JM (2006) Asymmetric coevolutionary networks facilitate biodiversity maintenance. Science 312: 431–433. 16627742 10.1126/science.1123412

[42] Zamora R (2000) Functional equivalence in plant-animal interactions: ecological and evolutionary consequences. Oikos 88: 442–447.

[43] Longstaff TG (1932) An ecological reconnaissance in West Greenland. Journal of Animal Ecology 1: 119–142.

[44] Elberling H, Olesen JM (1999) The structure of a high latitude plant-flower visitor system: the dominance of flies. Ecography 22: 314–323.

[45] Hawkins BA (1990) Global patterns of parasitoid assemblage size. Journal of Animal Ecology 59: 57–72.

[46] Böcher JJ (2001) Insekter og andre smådyr – i Grønlands fjeld og ferskvand. Nuuk: Atuagkat, 302 pp.

[47] McAlpine JF (1965) Insects and related terrestrial invertebrates of Ellef Ringnes Island. Arctic 18: 73–103.

[48] Olesen JM, Bascompte J, Dupont YL, Jordano P (2007) The modularity of pollination networks. Proceedings of the National Academy of Sciences of the United States of America 104: 19891–19896. 18056808 10.1073/pnas.0706375104 PMC2148393

[49] Elmendorf SC, Henry GHR, Hollister RD (2012) Global assessment of experimental climate warming on tundra vegetation: heterogeneity over space and time. Ecology Letters 15: 164–175. 22136670 10.1111/j.1461-0248.2011.01716.x

[50] Høye TT, Post E, Meltofte H, Schmidt NM, Forchhammer MC (2007) Rapid advancement of spring in the High Arctic. Current Biology 17: R449–R451. 17580070 10.1016/j.cub.2007.04.047

[51] Høye TT, Forchhammer MC (2008) Phenology of high-Arctic arthropods: effects of climate on spatial, seasonal and interannual variation. Advances in Ecological Research 40: 299–324.

