# Supplementary figures and images for: Strong Impact of Temporal Resolution on the Structure of an Ecological Network
Source: PLoS One. 2013 Dec 4;8(12):e81694. doi: 10.1371/journal.pone.0081694 (PMC3852737; doi:10.1371/journal.pone.0081694)

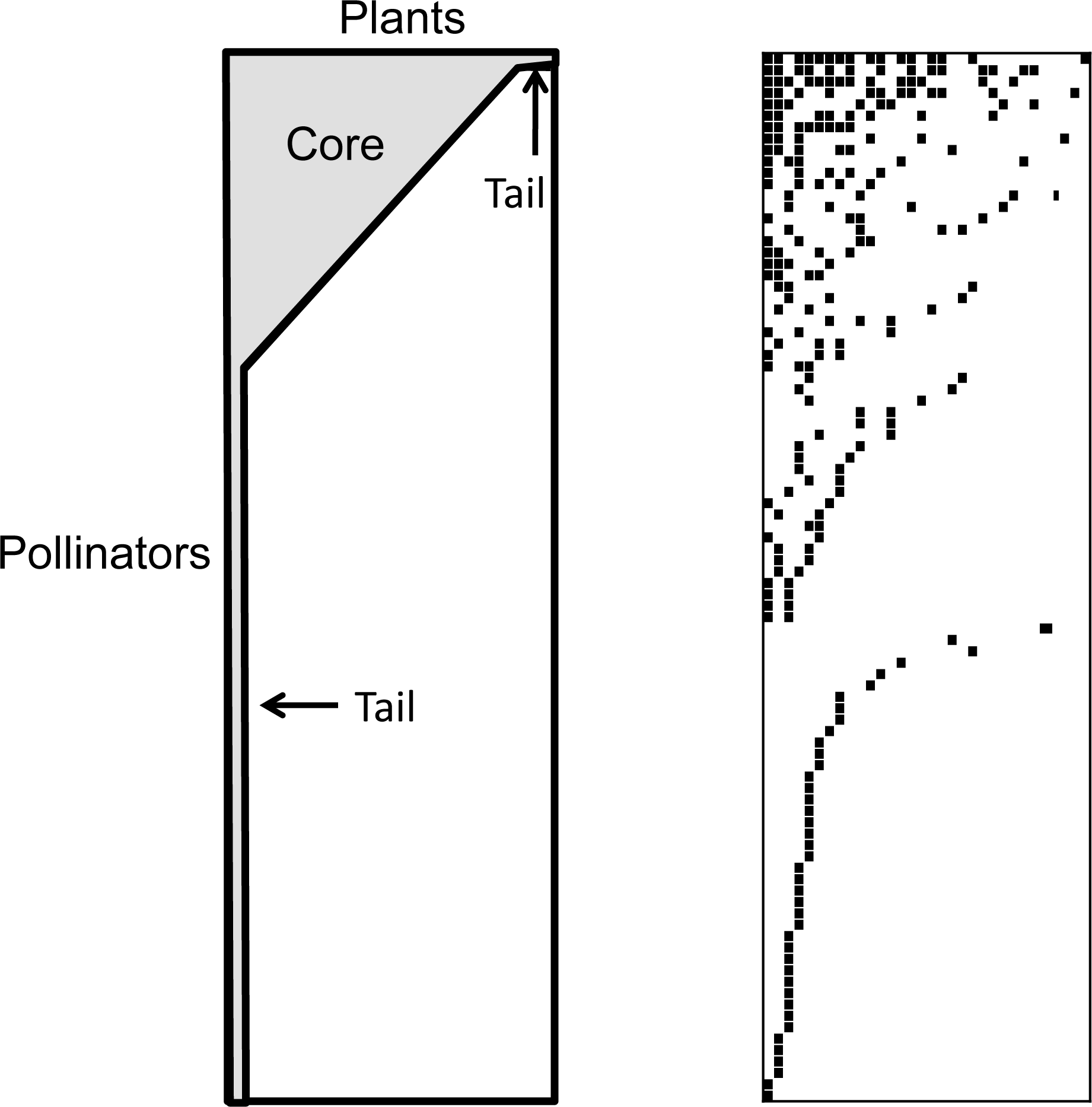

Supplement: File S1 — Matrices sorted in a nested way. Left: A perfectly nested matrix with all links in the shaded area. Right: 88 pollinators are listed in rows and 32 plants in columns (data from 2011). Species are listed according to descending linkage level L from the upper left corner. L of a species is its number of links to other species. If two species have similar L, they are subsequently sorted according to increasing L of their interacting partners. Sixty–one pollinator species (69% of total) and eight of all plant species (28%) constituted the tails. Thus the tail of the pollinator community is much longer than that of the plants. (TIF) [file pone.0081694.s001.tif]
