# Supplementary material for: Strong Impact of Temporal Resolution on the Structure of an Ecological Network
Source: PLoS One. 2013 Dec 4;8(12):e81694. doi: 10.1371/journal.pone.0081694 (PMC3852737; doi:10.1371/journal.pone.0081694)
Supplement: File S2 — Descriptive statistics from RangeModel analyses. For each core plant, we made 5,000 Monte Carlo runs, using the phenophases of tail pollinators. Empirical D is average daily difference between observed species number and mean species number from the Monte Carlo runs. Empirical rank is rank of the Empirical D compared to the 5,000 D-values from the runs. Percentile is Empirical rank/5,001. Mean, Min and Max D are average, minimum and maximum of the 5,000 runs. (DOCX) [file pone.0081694.s002.docx]

| **Core plant species** | **Study year** | **Empirical *D*** | **Empirical rank** | **Percentile** | **Mean *D*** | **Min *D*** | **Max *D*** |
| --- | --- | --- | --- | --- | --- | --- | --- |
| *Cerastium arcticum* | 2011 | 1.0538 | 4987 | 99.72 | 0.5924 | 0.2474 | 1.2062 |
| *Chamaenerion latifolium* | 2011 | 1.4084 | 5001 | 100.00 | 0.7451 | 0.3945 | 1.3873 |
| *Papaver radicatum* | 2011 | 1.3710 | 4872 | 97.42 | 0.7652 | 0.2657 | 1.9626 |
| *Saxifraga caespitosa* | 2011 | 1.6125 | 4995 | 99.88 | 0.8723 | 0.4213 | 1.7957 |
| *Dryas octopetala* | 2010 | 0.9848 | 4380 | 87.58 | 0.7227 | 0.3258 | 1.7535 |
| *Dryas octopetala* | 2011 | 1.4997 | 5001 | 100.00 | 0.6946 | 0.2980 | 1.4980 |
