# Supplementary material for: Strong Impact of Temporal Resolution on the Structure of an Ecological Network
Source: PLoS One. 2013 Dec 4;8(12):e81694. doi: 10.1371/journal.pone.0081694 (PMC3852737; doi:10.1371/journal.pone.0081694)
Supplement: File S3 — The importance of Muscidae and Anthomyiidae (Diptera) at Zackenberg. (DOCX) [file pone.0081694.s003.docx]

**S3. The importance of Muscidae and Anthomyiidae (Diptera) at Zackenberg**. At Zackenberg, many Muscidae were tail species (2010: 8 of 16 spp.; 2011: 10 of 16 spp.). The dominant genus was *Spilogona* with 13 species, and most of these were tail species (2010: 6 of 11 spp.; 2011: 8 of 11 spp.). These species constituted a seasonal sequence of flower visitors (C. Rasmussen *et al.* unpubl*.*). A second diverse Diptera family is the Anthomyiidae. Greenland has 32 species (V. Michelsen, pers. com.); eight were found at Zackenberg and most were tail species (2010: 5 of 6 spp.; 2011: 6 of 7 spp.). Their sole food as adults is nectar, except for cases of predatory feeding, e.g. in *Zaphne*, which may feed on Chironomidae (Griffiths 1997). The Zackenberg network had four *Zaphne* species, and all were tail species. If predators, these species belong partly to a higher trophic level together with hymenopteran parasitoids and hyperparasitoids (Várkonyi & Roslin 2013), and we expect such species to join the network later in the season when their prey become more abundant. In fact, the *Zaphne* species did so. In 2011, the season lasted from day 167 to day 235, and they did not link up to the network until at day 212, 214, and two on 231. The parasitic hymenopterans became more prominent in the latter half of the season, and especially in 2011, a pulse of late hymenopteran parasitoids was observed.

Griffiths, G.C.D. (1997). Anthomyiid flies (Diptera: Anthomyiidae) of the Yukon. In: *Insects of the Yukon* (eds. H.V. Danks & J.A. Downes). Biological surveys of Canada (Terrestrial arthropods), Ottawa, pp. 687–722.

Várkonyi, G. & Roslin, T. (2013). Freezing cold yet diverse: dissecting a high-Arctic parasitoid community associated with Lepidoptera hosts. The Canadian Entomologist, 145: 193-218.
